# Prevalence and predictors of androgen receptor and programmed death-ligand 1 in *BRCA1*-associated and sporadic triple-negative breast cancer

**DOI:** 10.1038/npjbcancer.2016.2

**Published:** 2016-02-24

**Authors:** Nadine Tung, Judy E Garber, Michele R Hacker, Vanda Torous, Gordon J Freeman, Emily Poles, Scott Rodig, Brian Alexander, Larissa Lee, Laura C Collins, Stuart J Schnitt

**Affiliations:** 1 Division of Hematology-Oncology, Beth Israel Deaconess Medical Center, Boston, MA, USA; 2 Harvard Medical School, Boston, MA, USA; 3 Division of Medical Oncology, Dana Farber Cancer Institute, Boston, MA, USA; 4 Department of Obstetrics and Gynecology, Beth Israel Deaconess Medical Center, Boston, MA, USA; 5 Department of Pathology, Beth Israel Deaconess Medical Center, Boston, MA, USA; 6 Department of Pathology, Brigham and Women’s Hospital, Boston, MA, USA; 7 Center for Immuno-Oncology, Dana-Farber Cancer Institute, Boston, MA, USA; 8 Department of Radiation Therapy, Brigham and Women’s Hospital, Boston, MA, USA

## Abstract

**Background:**

Triple-negative breast cancers comprise 15% of breast cancers and are more common in women with BRCA1 mutations. Although most have basal gene expression signatures, others resemble luminal tumors with expression of androgen receptor-related genes and some express the immunoinhibitory protein programmed death-ligand 1 (PD-L1). Given the availability of androgen receptor-targeted and immune therapies for triple-negative breast cancers, determining predictors of these biomarkers is important.

**Aims:**

To determine the prevalence and predictors of androgen receptor and PD-L1 expression in BRCA1-associated and sporadic triple-negative breast cancer.

**Methods:**

We studied 197 triple-negative breast cancers: 78 (39.6%) from BRCA1 mutation carriers and 119 (60.4%) from noncarriers. Tumor pathology was reviewed and tissue microarray sections were immunostained for androgen receptor and PD-L1.

**Results:**

Androgen receptor expression was seen in 18% of tumors and was significantly less common in tumors from BRCA1 mutation carriers than noncarriers (9.2 vs. 23.7%; *P*=0.01). Twenty-six percent of cancers expressed PD-L1 with no significant difference in frequency between carriers and noncarriers. Factors predicting androgen receptor expression were lower histologic grade (odds ratio (OR) 4.6; 95% confidence interval (CI) 1.1–19.7), older age at diagnosis (OR 1.3; 95% CI 1.03–1.7) and PD-L1 expression (OR 2.6; 95% CI 1.1–6.1). PD-L1 expression was significantly more common in cancers with lymphocytic infiltrates (OR, 3.3; 95% CI 1.1–10.4) and androgen receptor expression (OR, 3.2; 95% CI 1.4–7.5), and less common in tumors with lymphovascular invasion (OR 0.41; 95% CI 0.18–0.92).

**Conclusions:**

These results identify predictors for androgen receptor and PD-L1 expression among triple-negative breast cancers that may lead to better treatment selection and participation in clinical trials.

## Introduction

Triple-negative breast cancer (TNBC) comprises 15% of all breast cancers and is characterized by the absence of expression of estrogen receptor (ER), progesterone receptor (PR), and the human epidermal growth factor receptor 2 (HER2). TNBC is an aggressive subtype of breast cancer and its treatment is challenging in part owing to the heterogeneity of the disease. Recently, at least six molecular subtypes of TNBC have been identified by gene expression profiling with different clinical outcomes and responses to therapy.^1,2^

One subtype of TNBC is the luminal androgen receptor (LAR) group. While lacking the estrogen receptor, these TNBCs are heavily enriched in hormonally regulated pathways including androgen metabolism and androgen receptor (AR) signaling.^1,3,4^ LAR TNBCs express high levels of nuclear AR and luminal cytokeratins. There is some evidence that TNBCs that express the AR (i.e., AR+) are less sensitive to chemotherapy but can respond to AR antagonists.^2^ Six-month clinical benefit rates of 19% and 29% in metastatic AR+ TNBC have been reported with bicalutamide (TBCRC 011) and enzalutamide, respectively.^5,6^ A higher frequency of phosphatidylinositol-4,5-bisphosphate 3-kinase catalytic subunit alpha (PIK3CA) mutations has also been reported in the AR+ compared with other subtypes of TNBC suggesting another potential therapeutic strategy for these cancers.^7^

In addition, some TNBCs express programmed death-ligand 1 (PD-L1), a transmembrane protein expressed on both cancer cells and tumor-infiltrating inflammatory and immune cells. PD-L1 binding to programmed death 1 (PD-1) on T cells is one potential mechanism of tumor immune evasion.^8^ Recent successes with immune checkpoint inhibitors in TNBC have been reported. Overall response rates of 19% with pembrolizumab monotherapy and MPDL3280A (atezolizumab) monotherapy have been reported in advanced TNBCs that express PD-L1 (PD-L1+), with durable responses observed.^9,10^ Although responses to PD-1 and PD-L1 blockers are not restricted to tumors that express PD-L1, higher response rates have been observed in cancers with PD-L1 expression.^11–13^

Another group of TNBC can develop in women with germline *BRCA1* mutations. Seventy percent of the breast cancers that develop in women with inherited *BRCA1* mutations are TNBC and 10–20% of women with TNBC have a *BRCA1* mutation.^14–16^ Although outcomes after standard anthracycline-based adjuvant chemotherapy are similar for *BRCA1* mutation carriers and noncarriers with TNBC, the *BRCA1*-associated TNBCs appear to be more responsive to platinum chemotherapy, likely due to their defect in homologous recombination.^17–19^ It is not known whether the frequency of AR and PD-L1 expression differs among TNBC in women according to *BRCA1* status.

Owing to the heterogeneity of TNBC, identifying factors that predict for AR and PD-L1 expression and the association of a germline *BRCA1* mutation may facilitate the use of appropriate targeted therapies. To address this, we assessed the frequency of AR and PD-L1 expression in a cohort of primary TNBCs and determined whether the prevalence differed between TNBC from *BRCA1* mutation carriers (herein referred to as *BRCA1* carriers) and noncarriers. In addition, we evaluated whether any clinical or tumor pathologic features predicted for AR+, PD-L1+, or *BRCA1*-associated TNBC.

## Results

### Clinical and immunohistochemistry results

*BRCA1* mutation carriers were significantly younger at diagnosis than noncarriers (mean age 43.4 vs. 50.8 years; *P*<0.001). The most common histologic type was invasive ductal carcinoma among both carriers (92.2%) and noncarriers (90.2%). Compared with TNBC in noncarriers, *BRCA1*-associated TNBC significantly more often had high histologic grade as well as some degree of stromal lymphocytic infiltration (*P*=0.03). There was no difference in axillary nodal involvement between TNBC in *BRCA1* mutation carriers and noncarriers (Table 1).

The results of IHC staining of the tissue microarrays (TMAs) are presented in Table 2. CK5/6 expression was significantly more frequent in TNBC from *BRCA1* carriers than noncarriers (75.6% vs. 53.8%; *P*=0.002). There was no significant difference in CK14 or EGFR expression among TNBC in women with or without a *BRCA1* mutation.

### Androgen receptor expression

Among 194 TNBC with IHC staining results for AR, 35 (18.0%) expressed the AR, with at least 1% of cancer cells staining, whereas 22 (11.3%) demonstrated >10% of cancer cells staining. Compared with sporadic TNBC, *BRCA1*-associated TNBC significantly less often had any degree of AR expression (i.e., ⩾1% cells staining; 9.2% vs. 23.7%; *P*=0.01) or stronger AR expression (i.e., >10% cells staining; 3.9% vs. 16.1%; *P*=0.01: Table 2).

### PD-L1 expression

Of 193 TNBC evaluable for PD-L1 expression in cancer cells, 51 (26.4%) showed ⩾1% staining by IHC. There was no significant difference in PD-L1 cancer cell expression between TNBC from *BRCA1* carriers and noncarriers (22.7% vs. 28.8%; *P*=0.35). When PD-L1 positivity on cancer or inflammatory cells is considered, of the 177 TNBC evaluable, 163 (92.1%) were PD-L1+ with no significant difference in frequency between *BRCA1*-associated and sporadic TNBC (*P*=0.11: Table 2).

### Co-expression of AR and PD-L1

Of the 190 TNBCs for which both AR and PD-L1 staining results were available, 15 (7.9%) expressed both AR and PD-L1 in cancer cells by IHC. Of the 73 TNBC from *BRCA1* carriers, 3 (4.1%) expressed both, with one cancer having weak AR staining (1–10% cells) and 2 having >10% AR staining. Twelve (10.3%) of the 117 TNBC from noncarriers had co-expression of AR and PD-L1 on cancer cells, 5 with weak AR staining and 7 with >10% AR staining (data not shown).

### Logistic regression models for AR and PD-L1 expression and *BRCA1* status

Variables that were significantly associated with >10% AR expression by IHC staining in the multivariable model included older age (OR 1.3; 95% CI, 1.03–1.7 for every 5 years of age) and lower tumor grade (OR 4.6; 95% CI 1.1–19.7). In addition, PD-L1 positivity in cancer cells significantly predicted AR expression on ≥1% of cancer cells (OR= 2.6; 95% CI 1.1–6.1). In the multivariable model, after adjusting for age, tumor grade and PD-L1, *BRCA1* mutation status was no longer significantly associated with AR expression ⩾1 or >10% (Table 3).

Factors that significantly predicted PD-L1 expression in cancer cells in the multivariable model included the presence of a lymphocytic infiltration (OR 3.3; 95% CI 1.1–10.4), and ≥ 1% AR expression (OR 3.2; 95% CI 1.4–7.5) whereas the presence of lymphovascular invasion significantly decreased the odds that the cancer cells would express PD-L1 (OR 0.41; 95% CI 0.18–0.92: Table 4). No clinical features, tumor pathologic characteristic or IHC results significantly predicted for PD-L1 expression when PD-L1 positivity was defined as expression in cancer *or* inflammatory cells.

The three variables that were significantly more associated with having a *BRCA1* mutation in the multivariable model were younger age at diagnosis (OR=0.67; 95% CI 0.55–0.81 for every 5 years of age), presence of lymphocytic infiltration (OR= 3.0; 95% CI 1.1–8.0), and CK5/6 expression (OR= 3.0; 95% CI 1.4–6.4). While high histologic grade and lack of AR expression (<1% of cells staining) significantly predicted a *BRCA1* mutation on univariable analysis, they were not significant in the multivariable model (Supplementary Table 1).

## Discussion

The main findings from our study are that 18.0% of primary TNBCs express the AR and 11.3% have >10% cells staining. Although TNBCs from *BRCA1* carriers less frequently express the AR, on multivariable analysis the factors that predicted >10% AR expression were older age and lower grade, whereas PD-L1 expression on cancer cells significantly predicted ⩾1% AR expression. We also found that overall, 26.4% of TNBC expressed PD-L1 on cancer cells with no significant difference in frequency between *BRCA1*-associated and sporadic TNBC. The presence of lymphocytic infiltration and androgen receptor expression significantly predicted PD-L1 expression as did the absence of lymphovascular invasion.

Few studies have assessed the frequency of AR expression in early TNBC. In primary ER-negative breast cancer, the prevalence of AR expression has ranged from 10% to 32%,^20,21^ likely reflecting differences in techniques as well thresholds for considering a tumor AR+ (i.e., ⩾1% vs. >10% of cells staining). Gucalp *et al.*^5^ found 10% of 424 ER-negative breast cancers were AR+ (>10% cells staining) in the metastatic setting. Three other studies have evaluated AR positivity in TNBC specifically; McGhan *et al*.^22^ found 23% of 94 primary and recurrent TNBC expressed the AR (>10% staining). Luo *et al.*^23^ reported that 28% of 137 primary TNBC were AR+ (≥1% cells staining) and Collins *et al*.^24^ reported 31% AR positivity (>10% cells staining) in 216 primary basal breast cancers, defined as ER, PR and HER2-negative with either CK5/6 or EGFR expression by IHC staining. Our finding that 18% of primary TNBC express AR is slightly lower than other studies and may reflect that 40% of our cohort consisted of TNBC from *BRCA1* carriers for which the frequency of AR expression was lower. Our results are consistent with a smaller study that found that 21% of 30 *BRCA1*-associated ER-negative breast cancers expressed the AR using the same criteria of at least 1% of tumor nuclei staining.^25^

It is important to identify TNBC that express the AR as drugs targeting the AR are demonstrating activity. We found that older age and lower grade increased the likelihood that a TNBC expresses the AR. Although AR expression has been associated with lower proliferative rate^26^ and histologic grade^24^ among breast cancers this is the first study to our knowledge to report this finding in ER-negative breast cancers or TNBC specifically. McGhan *et al*.^22^ also found that older age predicted for AR expression in TNBC. Identifying older patients with TNBC who may respond to AR antagonists could be particularly important as they may not be optimal candidates for chemotherapy. Indeed, AR inhibitors are showing promise in ER-negative breast cancer. In a phase II trial using bicalutamide, a nonsteroidal antiandrogen, in women with AR+, ER− and PR-negative metastatic breast cancer, 19% of 21 evaluable patients achieved response or stable disease for at least 6 months.^5^ Similarly, a phase 2 study in advanced AR+ TNBC using enzalutamide, another AR antagonist, demonstrated a 29% 6-month clinical benefit rate in 75 evaluable patients.^6^ New agents such as selective androgen receptor modulators are also now in clinical trials. Although androgen receptor expression was significantly less common in TNBC from *BRCA1* carriers than noncarriers, this may reflect the younger age at diagnosis and higher grade of *BRCA1*-associated TNBC, as *BRCA1* mutation status was no longer significant on multivariable analysis.

Our finding that 26% of primary TNBC express PD-L1 on the cancer cell surface is consistent with the findings of Mittendorf *et al*.^27^ who reported that 19% of 105 primary TNBC showed cancer cell PD-L1 positivity. We used the 9A11 mouse monoclonal antibody that recognizes an epitope in the cytoplasmic domain of PD-L1 and gives good membrane staining; Mittendorf *et al*. used the 5H1 antibody that recognizes an extracellular epitope. One conundrum is that different studies have used different criteria for considering a tumor PD-L1+. There is no consensus regarding whether cancer cells, inflammatory infiltrates or both should be considered; the threshold for PD-L1 positivity; whether to use qualitative or quantitative methods; or the optimal antibody. When PD-L1 positivity was defined as ⩾1% of cancer or inflammatory cells staining, 92% of all TNBC in our cohort were considered PD-L1+, making this cutoff value less useful. Other studies have reported PD-L1 positivity in 30–45% of breast cancers by varying criteria of epithelium and stromal cell staining using quantitative analysis of various monoclonal antibodies, and have reported that expression is higher in TNBC.^28,29^

Since PD-L1 expression enriches for response to anti-PD1 and anti-PD-L1 agents, it is useful to identify predictors of PD-L1 expression among TNBC.^11–13^ We found that the presence of lymphocytic infiltration significantly predicted PD-L1 expression on cancer cells. Denkert *et al.*^30^ also found that mRNA expression of 12 immune-related genes, including PD-L1 was higher among 314 TNBC with higher levels of stromal tumor-infiltrating lymphocytes (TILs) in the GeparSixto trial. Wimberly *et al.*^28^ found a positive correlation between TILs and PD-L1 expression in 105 primary breast cancers and Mittendorf *et al*.^27^ found that CD8+ T cells were more frequent in PD-L1+ versus PD-L1-negative TNBC. PD-L1 expression may be expressed constitutively on cancer cells or may be induced by cytokines such as gamma interferon secreted by activated T cells.^31^ Indeed, classification of tumors into four groups based on the presence or absence of TILs and PD-L1 expression on cancer cells has been proposed to guide immune therapy approaches.^32^ It is likely that further characterization of the subset of TILs will also be important in planning tumor specific immune approaches.

As lymphocytic infiltration was significantly more common in TNBC from *BRCA1* carriers than noncarriers, one might have expected that *BRCA1*-associated TNBC also would have a higher frequency of PD-L1 expression. We did not find this to be true. It has been proposed that tumors with higher somatic mutational burdens may be those that are more likely to induce an immune response and respond to immune therapy as a result of neoantigen creation. Recently, Le *et al*.^33^ reported that colorectal and other cancers with germline or somatic loss of DNA mismatch repair (MMR) genes have a significantly higher response rate and 12-week clinical benefit rate to pembrolizumab than MMR-proficient colorectal tumors. It is possible that as the somatic mutational signature induced by *BRCA1* mutations is different than that associated with MMR deficiency, the nature of the immune response induced is also different.^34^

We found that TNBCs that express the AR are also threefold more likely to express PD-L1 on cancer cells. Overall, 7.9% of the TNBC in our cohort coexpressed AR and PD-L1 with 10.3% of TNBC from noncarriers demonstrating expression of both receptors. If confirmed, this could provide the basis for combination therapy with AR inhibitors and immune therapy for this subset of TNBC.

It is useful to identify patients with TNBC who have a *BRCA1* mutation as therapies such as PARP inhibitors and platinum agents are more active in this subset of TNBC.^19,35–37^ The National Comprehensive Cancer Network now recommends genetic testing for any woman diagnosed with TNBC by age 60 years.^38^ However, identifying *BRCA1*-associated TNBC by pathologic features and/or biomarker expression may increase recognition of these tumors. We found that younger age at diagnosis as well as lymphocytic infiltration and expression of the basal cytokeratin 5/6 in the tumor significantly increased the likelihood that a TNBC was associated with a *BRCA1* mutation. Our findings are consistent with other studies that demonstrated that younger age^15,16^ and basal cytokeratin expression^39^ are significantly more common in *BRCA1*-associated than sporadic TNBC. To our knowledge, this is the first report that among TNBC, lymphocytic infiltration is significantly more common in *BRCA1*-associated cancers.

A potential limitation of this study is that the tumor present on cores in the TMA may not be representative of the entire tumor if particular cancers are heterogeneous. In addition, assessment of immunostains in this study was qualitative, which introduces the possibility of inter-pathologist variability. However, the extent of any variability should differ with respect to any of the outcomes assessed in this study. In addition, if qualitative interpretation of AR and PD-L1 stains can be standardized, as they have been for steroid receptors and HER2, then more widespread use of these biomarkers may be feasible.

In conclusion, our study confirms the heterogeneity of TNBC and contributes to understanding the factors that increase identification of various subtypes of TNBC. Germline *BRCA1* mutation status, AR and PD-L1 expression all provide important information that predicts response to an increasing number of targeted therapies. Confirmatory studies will be important and may lead to better selection of patients with TNBC for treatment and participation in clinical trials.

## Materials and methods

Two hundred thirty Stage I-III TNBC diagnosed from 1989 through 2008 from women who had *BRCA1* testing were identified through the clinical databases and annotated Specialized Program of Research Excellence (SPORE) specimen bank at Beth Israel Deaconess Medical Center and Dana–Farber Cancer Institute. ER, PR, and HER2 status, assessed as part of the routine clinical evaluation, was abstracted from institutional pathology reports. We excluded 9 TNBCs from *BRCA2* mutation carriers, 3 TNBCs for which a previous cancer from that individual was included and 21 cancers with insufficient remaining tumor for research use. The final study cohort, therefore, consisted of 78 TNBC from *BRCA1* carriers and 119 TNBC from noncarriers. *BRCA1* mutation status was established for 120 of these women by high-throughput heteroduplex detection from a banked research blood sample as described previously.^40^ The other 77 women had genetic testing through commercial laboratories through high-risk clinics.

Clinical characteristics were abstracted from medical records, and included age at diagnosis and clinical stage (T size, N stage). Histologic sections of *BRCA1*-associated and sporadic TNBC were reviewed by the two study pathologists (SJS and LC). Each cancer was scored for the following pathologic features: histologic type; Nottingham combined histologic grade, with each of the three components of grade (i.e., tubule formation, nuclear grade, and mitotic rate) recorded separately; extent of stromal lymphocytic infiltrate (i.e., negative, focally positive, or positive); and presence of lymphovascular invasion.

**Figure 1:**
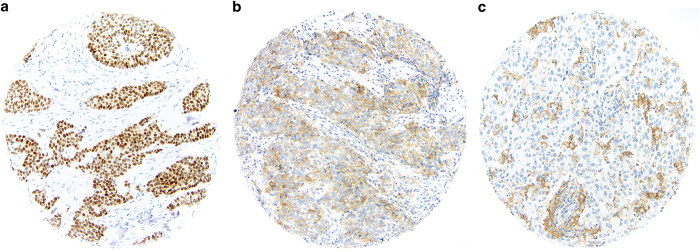
Triple-negative breast cancers showing (**a**) nuclear expression of androgen receptor; (**b**) tumor cell staining for PD-L1; (**c**) immune cell staining for PD-L1.

TMAs were constructed in the Dana–Farber Harvard Cancer Center Tissue Microarray Core Facility by obtaining three 0.6-mm cores from each tumor after study pathologists identified representative areas of invasive cancer. Sections cut from the tissue microarrays were immunostained using mouse monoclonal antibodies for basal cytokeratins (CK5/6, CK14), epidermal growth factor receptor (EGFR), AR and the 9A11 mouse monoclonal antibody to PD-L1 (developed by GJF;^41^ Table 5). For all cytokeratins and EGFR, staining was scored as either negative (no tumor cell staining) or positive (at least 1% tumor cells staining). For AR expression, staining was scored as negative (no cancer cell nuclear staining), weakly positive (1–10% cancer cell nuclei staining), or positive (>10% cancer cell nuclei staining). For PD-L1 expression immunohistochemical (IHC) staining was assessed in both cancer cells and inflammatory cells. PD-L1 positivity (PD-L1+) was defined as at least 1% of cells staining (Figure 1). We classified a case as positive if there was staining in any of the three cores from that case.

Descriptive data are presented as mean±s.d., median (interquartile range), or frequency and proportion. Continuous data were compared using a *t*-test or Wilcoxon rank sum test, depending on data distribution, and categorical data were compared with the *χ*^2^ or Fisher’s exact test. We used logistic regression to calculate the odds ratio (OR) and 95% confidence interval (CI) and used Firth estimation to deal with sparse data, as necessary.^42^ The outcomes assessed were *BRCA1* carrier status, PD-L1 cancer positivity and AR staining with two cutoffs for positivity: ⩾1 and >10%. For *BRCA1* carrier status, PD-L1 cancer positivity and AR staining ⩾1%, we built a multivariable logistic regression model including all predictor variables that were significantly associated with the outcome in the crude models. For AR staining >10% we used the same variables as in the multivariable model for AR staining ⩾1%. All tests were two-sided and a *P*<0.05 was considered statistically significant. All statistical analyses were conducted with SAS 9.4 (SAS Institute, Cary, NC, USA).

This study was approved by the institutional review board of Dana Farber-Harvard Cancer Center (DF-HCC 07-334). Given the retrospective nature of the study, subject consent was not required.

## Figures and Tables

**Table 5:** Antibodies and dilutions used

*Antibody to*	*Clone*	*Manufacturer*	*Dilution*
Androgen receptor	AR441	Dako	1:200
PD-L1	405.9A11	Freeman lab	1 to 125 10.4 μg/ml
Cytokeratin 5/6	D5/16B4	Dako	1:50
Cytokeratin 14	LL02	NeoMarkers	1:200
Epidermal Growth Factor receptor	2–18C9 Prediluted	Dako	Prediluted (pharmDX kit)

**Table 1:** Clinical and pathological features at presentation

	*BRCA1 Carrier (*n*=78)*	*Noncarrier (*n*=119)*	P-*value*
Age at diagnosis (years)—mean±s.d.^a^	43.4±8.8	50.8±10.8	<0.001
Histology—*n* (%)			0.94
Ductal	71 (92.2)	101 (90.2)	
Lobular	0 (0.0)	1 (0.9)	
Mixed ductal/lobular	5 (6.5)	9 (8.0)	
Metaplastic	1 (1.3)	1 (0.9)	
Unknown	1	7	
Tumor size (cm)—median (IQR)^a^	1.7 (1.2–2.2)	2.0 (1.4–3.0)	0.06
Tumor grade—*n* (%)			0.03
1	0 (0.0)	1 (0.9)	
2	1 (1.3)	11 (9.6)	
3	77 (98.7)	103 (89.6)	
Unknown	0	4	
Lymphovascular invasion—*n* (%)			0.54
Present	26 (33.3)	44 (37.6)	
Absent	52 (66.7)	73 (62.4)	
Unknown	0	2	
Lymphocytic infiltrate—*n* (%)			0.03
Negative	8 (10.4)	27 (24.3)	
Focally positive	42 (54.5)	58 (52.3)	
Positive	27 (35.1)	26 (23.4)	
Unknown	1	8	
Positive lymph nodes—*n* (%)			0.81
Present	32 (45.7)	43 (43.9)	
Absent	38 (54.3)	55 (56.1)	
Unknown	7	21	
T classification—*n* (%)			0.03
T1	56 (72.7)	54 (53.5)	
T2	20 (26.0)	39 (38.6)	
T3	1 (1.3)	5 (5.0)	
T4	0 (0.0)	3 (3.0)	
Unknown	1	18	
N classification—*n* (%)			0.99
N0	38 (54.3)	55 (56.1)	
N1	23 (32.9)	30 (30.6)	
N2	7 (10.0)	10 (10.2)	
N3	2 (2.9)	3 (3.1)	
Unknown	8	21	

Abbreviation: IQR, interquartile range.

a Age at diagnosis and tumor size are missing for 1 carrier and 18 noncarriers.

**Table 2:** Tissue microarray immunohistochemistry results

	*BRCA1 Carrier (*n*=78)*	*Noncarrier (*n*=119)*	P-*value*
EGFR—*n* (%)			0.10
Negative	14 (18.9)	35 (29.7)	
Positive	60 (81.1)	83 (70.3)	
Unknown^a^	4	1	
Cytokeratin 5/6—*n* (%)			0.002
Negative	19 (24.4)	55 (46.2)	
Positive	59 (75.6)	64 (53.8)	
Unknown^a^	0	0	
Cytokeratin 14—*n* (%)			0.42
Negative	37 (48.7)	65 (54.6)	
Positive	39 (51.3)	54 (45.4)	
Unknown^a^	2	0	
Androgen receptor—*n* (%)			0.02
Negative	69 (90.8)	90 (76.3)	
Weakly positive	4 (5.3)	9 (7.6)	
Positive	3 (3.9)	19 (16.1)	
Unknown^a^	2	1	
Androgen receptor—*n* (%)			0.01
Negative	69 (90.8)	90 (76.3)	
Weakly positive/positive (⩾1%)	7 (9.2)	28 (23.7)	
Unknown^a^	2	1	
Androgen receptor—*n* (%)			0.01
Negative/weakly positive	73 (96.1)	99 (83.9)	
Positive (>10%)	3 (3.9)	19 (16.1)	
Unknown^a^	2	1	
PD-L1 cancer—*n* (%)			0.35
Negative	58 (77.3)	84 (71.2)	
Positive (⩾1%)	17 (22.7)	34 (28.8)	
Unknown^a^	3	1	
PD-L1 cancer/inflammatory—*n* (%)			0.17
Negative^b^	3 (4.3)	11 (10.3)	
Positive (⩾ 1%)^c^	67 (95.7)	96 (89.7)	
Unknown	8	12	

a Insufficient measurable tumor.

b Cancer cells and inflammatory cells lack PD-L1 staining.

c Either cancer cells or inflammatory cells stain for PD-L1.

**Table 3:** Logistic regression models predicting androgen receptor expression by IHC stains

	*Androgen receptor staining ⩾1%*		*Androgen receptor staining >10%*	
	*Crude odds ratio (95% CI)*	*Adjusted odds ratio*^a^ *(95% CI)*	*Crude odds ratio (95% CI)*	*Adjusted odds ratio*^a^ *(95% CI)*
*BRCA1 mutation status*				
Carrier versus noncarrier	0.33 (0.14–0.79)	0.47 (0.17–1.3)	0.21 (0.06–0.75)	0.43 (0.11–1.7)
				
5-year change in age at diagnosis	1.2 (1.04–1.5)	1.2 (0.97–1.4)	1.4 (1.1–1.7)	1.3 (1.03–1.7)
				
*Histology*				
Ductal versus mixed	1.2 (0.26–5.8)		0.65 (0.13–3.2)	
				
*Tumor grade*				
1/2 vs. 3	6.5 (2.0–20.9)	3.8 (0.98–14.6)	6.3 (1.9–21.6)	4.6 (1.1–19.7)
				
*Lymphovascular invasion*				
Present versus absent	1.1 (0.52–2.4)		0.84 (0.33–2.2)	
				
*Lymphocytic infiltrate*				
Positive/focally positive versus negative	0.70 (0.29–1.7)		0.53 (0.19–1.5)	
				
*EGFR*				
Positive versus negative	0.77 (0.34–1.8)		1.2 (0.41–3.4)	
				
*Cytokeratin 5/6*				
Positive versus negative	0.77 (0.36–1.6)		0.69 (0.28–1.7)	
				
*Cytokeratin 14*				
Positive versus negative	0.52 (0.24–1.1)		0.48 (0.19–1.2)	
				
*PD-L1 cancer*				
Positive versus negative	2.7 (1.3–5.9)	2.6 (1.1–6.1)	2.3 (0.92–6.0)	2.8 (0.98–7.8)
				
*PD-L1 cancer/inflammatory*				
Positive versus negative	1.4 (0.30–6.7)		1.8 (0.23–14.9)	

Abbreviations: CI, confidence interval; IHC, immunohistochemical.

a Adjusted for all other variables that were significantly associated with androgen receptor staining.

**Table 4:** Logistic regression models predicting PD-L1 cancer expression by IHC stains

	*Crude odds ratio (95% CI)*	*Adjusted odds ratio*^a^ *(95% CI)*
5-year change in age at diagnosis	0.98 (0.83–1.1)	
*Histology*		
Ductal versus mixed	11.4 (0.60–214.6)	
		
*Tumor grade*		
3 vs. 1/2	0.72 (0.21–2.5)	
		
*Lymphovascular invasion*		
Present versus absent	0.40 (0.19–0.85)	0.41 (0.18–0.92)
		
*Lymphocytic infiltrate*		
Positive/focally positive versus negative	3.1 (1.0–9.4)	3.3 (1.1–10.4)
		
*EGFR*		
Positive versus negative	1.6 (0.71–3.6)	
		
*Cytokeratin 5/6*		
Positive versus negative	1.0 (0.53–2.0)	
		
*Cytokeratin 14*		
Positive versus negative	0.93 (0.49–1.8)	
		
*Androgen receptor*		
⩾1% vs. <1%	2.7 (1.3–5.9)	3.2 (1.4–7.5)

Abbreviation: IHC, immunohistochemical.

a Adjusted for all other variables that were significantly associated with PD-L1 cancer.
