# Supplementary material for: Prevalence and predictors of androgen receptor and programmed death-ligand 1 in BRCA1-associated and sporadic triple-negative breast cancer
Source: NPJ Breast Cancer. 2016 Feb 24;2:16002–. doi: 10.1038/npjbcancer.2016.2 (PMC5515333; doi:10.1038/npjbcancer.2016.2)
Supplement: Supplementary Table 1 [file npjbcancer20162-s1.doc]

**Supplemental Table 1**: Logistic regression models predicting BRCA1 mutation carrier status

|  | **Crude odds ratio**  **(95% CI)** | **Adjusted odds ratio***  **(95% CI)** |
| --- | --- | --- |
| **5-year change in age at diagnosis** | **0.68 (0.58-0.81)** | **0.67 (0.55-0.81)** |
| **Histology** |  |  |
| **Ductal vs. mixed** | **1.3 (0.41-3.9)** |  |
| **Tumor grade** |  |  |
| **3 vs. 1/2** | **9.0 (1.1-70.5)** | **19.5 (0.89-428.4)** |
| **Lymphovascular invasion** |  |  |
| **Present vs. Absent** | **0.83 (0.46-1.5)** |  |
| **Lymphocytic infiltrate** |  |  |
| **Positive/focally positive vs. Negative** | **2.8 (1.2-6.5)** | **3.0 (1.1-8.0)** |
| **EGFR** |  |  |
| **Positive vs. Negative** | **1.8 (0.89-3.7)** |  |
| **Cytokeratin 5/6** |  |  |
| **Positive vs. Negative** | **2.7 (1.4-5.0)** | **3.0 (1.4-6.4)** |
| **Cytokeratin 14** |  |  |
| **Positive vs. Negative** | **1.3 (0.71-2.3)** |  |
| **Androgen receptor** |  |  |
| **<1% vs. ≥1%** | **3.1 (1.3-7.4)** | **2.8 (0.95-8.4)** |
| **PD-L1 cancer** |  |  |
| **Positive vs. Negative** | **0.72 (0.37-1.4)** |  |
| **PD-L1 cancer/inflammatory** |  |  |
| **Positive vs. Negative** | **2.6 (0.69-9.5)** |  |

***Adjusted for all other variables that were significantly associated with BRCA1 carrier status**
